# Fenofibrate Treatment Enhances Antioxidant Status and Attenuates Endothelial Dysfunction in Streptozotocin-Induced Diabetic Rats

**DOI:** 10.1155/2010/828531

**Published:** 2010-12-27

**Authors:** Murat Olukman, Ebru Demirel Sezer, Sibel Ülker, Eser Y. Sözmen, Gülcihan Mehtap Çınar

**Affiliations:** ^1^Department of Medical Pharmacology, Faculty of Medicine, Ege University, Bornova, 35100 İzmir, Turkey; ^2^Department of Medical Biochemistry, Faculty of Medicine, Ege University, Bornova, 35100 İzmir, Turkey

## Abstract

Diabetic endothelial dysfunction is accompanied by increased oxidative stress and upregulated proinflammatory and inflammatory mediators in the vasculature. Activation of peroxisome proliferator-activated receptor-alpha (PPAR-**α**) results in antioxidant and anti-inflammatory effects. This study was designed to investigate the effect of fenofibrate, a PPAR-**α** activator, on the endothelial dysfunction, oxidative stress, and inflammation in streptozotocin diabetic rats. Diabetic rats received fenofibrate (150 mg kg^−1^ day^−1^) for 4 weeks. Fenofibrate treatment restored the impaired endothelium-dependent relaxation and increased basal nitric oxide availability in diabetic aorta, enhanced erythrocyte/liver superoxide dismutase and catalase levels, ameliorated the abnormal serum/aortic thiobarbituric acid reactive substances, and prevented the increased aortic myeloperoxidase without a significant change in serum total cholesterol and triglyceride levels. It did not affect the decreased total homocysteine level and the increased tumor necrosis factor-**α** level in the serum of diabetic rats. Fenofibrate-induced prevention of the endothelial function seems to be related to its potential antioxidant and antiinflammatory activity.

## 1. Introduction

Diabetes Mellitus (DM) is a common, chronic group of metabolic diseases which is associated with various vascular, neuronal, endocrine, and immune alterations at cellular, tissues and organ levels [1]. Macro- and microvascular disease, are currently accepted to be the most common determinants of morbidity and mortality in the course of DM [2]. However, the exact mechanisms of the development of vascular disease have not been completely elucidated until now.

Endothelial dysfunction and oxidative stress play a key role in the pathogenesis of diabetic vascular disease [3]. Under physiological conditions, vascular endothelium plays an important role in the formation of vascular smooth muscle tone by releasing relaxant mediators, such as nitric oxide (NO), endothelium derived hyperpolarizing factor, endothelium derived contracting factor, and prostacycline [4]. Impairment of endothelium-dependent relaxation has been demonstrated both in vessels of patients with insulin-dependent diabetes mellitus [5] and noninsulin-dependent diabetes mellitus [6]. Although the reason for this impairment is not entirely clear, some theories have been postulated. As is known, DM is characterized by hyperglycemia and hyperlipidemia, two cardinal biochemical features associated with inhibition of endothelial nitric oxide synthase (eNOS), leading to diminished NO production and increased formation of reactive oxygen species (ROS) in endothelial and vascular smooth muscle cells. Besides, impaired expression or activity of some antioxidant enzymes such as superoxide dismutase (SOD) and catalase contributes to the development of endothelial dysfunction in DM by increasing oxidative stress [7]. 

Endothelial dysfunction accompanied by upregulated proinflammatory and inflammatory mediators is thought to be another contributing factor to the pathogenesis of diabetic vascular complications. Multiple effects of inflammatory cytokines like interleukin-1 (IL-1) and tumor necrosis factor-*α* (TNF-*α*), which lead to prothrombotic and proinflammatory changes on the vascular endothelium, have been outlined in some reports [8]. Recently it has been suggested that myeloperoxidase (MPO), a “heme” protein derived from leukocytes, plays an important role in leukocyte-mediated vascular injury responses in inflammatory vascular diseases such as diabetic vasculopathy and atherosclerosis [9, 10]. 

In diabetic patients, hyperhomocysteinemia is an independent risk factor for macroangiopathy and mortality [11]. Many ROS are generated during the auto-oxidation of homocysteine (Hcy) in diabetic patients. These oxygen-derived molecules initiate the lipid peroxidation in cell membranes that are responsible for endothelial injury and reduction of vascular NO production [12].

Peroxisome proliferator-activated receptor-*α* (PPAR-*α*) is a hormonal activated nuclear receptor which plays an important role in the course of many vascular diseases such as DM, hypertension, and coronary heart disease [13, 14]. In recent publications, it has been clearly demonstrated that activation of PPAR-*α* leads to an antiinflammatory effect by reducing plasma concentrations of TNF-*α*. On the other hand, it produces an antioxidant effect by reducing plasma concentrations of malonyldialdehyde, major indicator of oxidative stress, and by stimulating the expression of SOD, one of the major molecules of antioxidant defense [15, 16]. In this context fenofibrate (FF), a third generation fibric acid derivative and a PPAR-*α* agonist, can be a beneficial choice for the treatment of diabetic vascular complications because of its antiinflammatory and antioxidant effects. Moreover, FF is a useful drug for the treatment of atherogenic dyslipidemias, producing a substantial decrease in the levels of triglyceride-rich lipoproteins and an increase in high density lipoprotein cholesterol levels. In contrast, several studies show that FF can significantly increase plasma Hcy levels [17, 18]. However, the underlined mechanisms by which FF increase total Hcy levels and whether they have any adverse effects on endothelial function are unknown. 

In light of the foregoing data, the primary aim of this study is to investigate the role of FF on diabetic endothelial dysfunction, to elucidate its antioxidant and anti-inflammatory effects, and to evaluate the contribution of FF-induced hyperhomocysteinemia in diabetic vascular complications in a rat model of streptozotocin- (STZ-) induced DM. As far as we know this is the first study in literature which discusses all these parameters in one single study protocol.

## 2. Materials and Methods

### 2.1. Animals

Male Wistar rats (body weight 200–250 g, 10–12 weeks old) were used in this study. The animals were housed in individual cages at a constant temperature (22°C) with a fixed 12 : 12-h light-dark cycle. This study was approved by the Ethics Committee of the School of Medicine, University of Ege (Approval no: 2004-17).

The animals were randomised to four experimental groups: untreated control, untreated diabetic, FF-treated diabetic (150 mg kg^−1^ day^−1^ via oral gavage), and FF-treated control. Diabetes was induced by a single intraperitoneal injection of STZ (55 mg kg^−1^). STZ was dissolved in 1 ml cold fresh saline immediately before use. Seven days after STZ injection, blood glucose levels were determined using an Accu-Chek Go glucometer (Roche, Turkey). Rats with blood glucose levels of 250 mg dl^−1^ or above were considered to be diabetic. Control rats were injected intraperitoneally with 1 ml cold fresh saline. FF treatment was started 6 weeks after STZ or saline injection, and the treatment continued for 4 weeks.

### 2.2. Vascular Reactivity Studies

10 weeks after STZ or saline injection, rats were killed by the withdrawal of blood via cardiac puncture under anesthesia. Thoracic aortas were quickly removed into 4°C Krebs-Henseleit solution and cut into four 3-4 mm wide rings. In some rings, the endothelium was quickly removed mechanically by inserting a small forceps into the lumen and gently rolling. In each experiment, endothelium-intact and endothelium-denuded rings (two of each) were suspended horizontally under a resting tension of 2 g in 20 ml organ chambers containing Krebs-Henseleit solution of the following composition (mM): NaCl, 118.30; KCl, 4.70; MgSO_4_, 1.20; KH_2_PO_4_, 1.22; CaCl_2_, 2.50; NaHCO_3_, 25.00; glucose, 11.10; ph, 7.4; gassed with a 95% O_2_, 5% CO_2_ mixture and maintained at 37°C. Each ring was connected to a force displacement transducer for the measurement of isometric force which was continuously displayed and recorded online on a personal computer via an 8-channel transducer data acquisition system (BIOPAC COMMAT İletişim Ltd., Ankara, Turkey) using a software (BIOPAC MP35 COMMAT İletişim Ltd., Ankara, Turkey) which also analysed the data.

After 15 min of equilibration, each ring was systematically stretched to the optimum of its length-active tension relation by exposure to incremental concentrations of KCl. Rings were then left to equilibrate in the bath for a total of 30 min and washed every 15 min. After the initial equilibration period of 60–90 min, endothelium-denuded rings were used to assess the contractile responses elicited by either incremental concentrations of phenylephrine (PE) (0.001–30 *μ*M) or a single concentration of KCl (120 mM). 

Relaxant responses were determined using cumulative concentrations of acetylcholine (0.001–30 *μ*M, ACh), calcium ionophore A23187 (0.001–3 *μ*M, A23187), L-arginine (0.1–300 *μ*M), or sodium nitroprusside (0.0001–0.3 *μ*M, SNP) on endothelium-intact rings precontracted with submaximal concentration of phenylephrine. In order to maintain appropriate precontractile tension in all preparations, submaximal concentrations were determined using the prior cumulative concentration-response curves of PE. Contractile responses generated by cumulative concentrations of PE (0.001–30 *μ*M) were also assessed in these rings. 

In endothelium-intact rings, the production of basal NO was also evaluated by calculating the ratio of additional contractions induced by a single concentration of NG-Nitro-L-arginine methyl ester (100 *μ*M, L-NAME) to the precontraction elicited by a single concentration of PE given at median effective concentration (EC_50_).

### 2.3. Biochemical Measurements

After obtaining heparinized blood samples by intracardiac puncture, serum was immediately separated, and erythrocyte hemolyzates were prepared. For this purpose, the packed erythrocytes were washed two times with 9 g/L NaCI solution and haemolysed with ice-cold water (1/5, v/v). Erythrocyte SOD and catalase activities were determined immediately in hemolyzates. The haemoglobin values were measured by Drabkin's method. The erythrocyte SOD activities were measured based on the inhibition of autoxidation of epinephrine by SOD at 480 nm, with an LKB Ultraspec 2 spectrophotometer (LKB Biocrom Ltd, Cambridge, England). The assay was calibrated by using purified SOD, and done unit of enzyme was defined as the amount of enzyme, which inhibits 50% of autoxidation of epinephrine. The erythrocyte catalase activities were determined as described by Sözmen et al. [19]. According to this method, the degradation of hydrogen peroxide is recorded spectrophotometrically at 240 nm absorbance. One unit of catalase is defined as the amount of enzyme, which decomposes 1 *μ*mol hydrogen peroxide/min under specific conditions.

In addition, aortic and liver tissue samples were homogenized in phosphate buffer (0.5 M; pH = 7.0), (1/10 w/v). The homogenate was centrifuged for 5 min at 700 × g at 4°C to sediment unbroken cells and cellular debris. Determination of SOD and catalase activities in the supernatants, as defined above, and the determination of lipid peroxidation were carried out immediately. Lipid peroxidation was briefly measured by the determination of thiobarbituric acid reactive substances (TBARSs) which was performed by the incubation of tissue homogenates in TBARS solution (0,12 M thiobarbituric acid in 15% trichloroacetic acid and 1% hydrochloric acid mixture) for 30 min at 95°C. TBARS levels were calculated using 1, 1, 3, 3 tetramethoxypropane standard curve. All biochemical parameters were normalized to total protein content of the heamolyzate as measured by the Lowry method using bovine-serum albumin as standard [20].

### 2.4. Measurement of MPO and TNF-*α* Levels

Tissue MPO activities were measured according to the modified method of Grisham et al. [21]. Briefly, following homogenization of aortic tissue, homogenates were centrifuged at 10000 rpm for 15 min. Pellets were rehomogenized in 0.5 mM HETAB (hexadecyltrimethyl ammonium bromide) in phosphate buffer (50 mM, pH = 6.0). Following three freeze and thaw cycles, samples were centrifuged at 10000 rpm for 10 min. Supernatants were added to reactive solution containing 0.5 M o-dianisidin (in phosphate buffer). After addition of hydrogen peroxide solution (20 mM), absorbance of samples was recorded at 492 nm with a microplate reader for 3 minutes with 15-second intervals. MPO activities were calculated using a standard curve.

The serum content of TNF-*α* was determined spectrophotometrically according to the instructions of a commercially available ELISA kit (Rat TNF ELISA Kit BD Biosciences Inc.).

### 2.5. Measurement of Serum Total Cholesterol and Triglyceride Levels

Lipid levels were measured in the fasting serum samples by using ready-made cholesterol kits (Cormary Lot: 304-1910 A Lublin Poland) and triglyceride kits (Cormary Lot 301-141409 Lublin Poland) on the Hitachi 912 Automatic Analyzer machine.

### 2.6. Measurement of Serum Hcy Levels

Blood samples obtained by cardiac puncture were centrifuged (1000 × g for 10 min), and serum was separated. Serum total Hcy was measured by Fluorescence Polarization Immunoassay using an IMx automatic analyser (Abbott Laboratories, Diagnostic Division, Abbott Park, IL, USA).

### 2.7. Drugs

Streptozotocin, acetylcholine, phenylephrine, L-arginine, calcium ionophore A23187, sodium nitroprusside, and _L_-NAME were obtained from Sigma Chemical Co. (St. Louis, MO, USA). FF was supplied from Nobel Drug Co. (Interlab Ltd, Turkey).

### 2.8. Statistical Analysis

Data were given as mean ± SEM. Concentration response curves were configured with nonlinear regression analysis prior to the evaluation of EC_50 _and *p*D_2_ (−log EC_50_) via Graphpad Prism 4.0 program. The contractile response of each aortic ring to phenylephrine was recorded in milligrams by using BIOPAC Mp35 software program. Relaxation responses were evaluated as percentage of phenylephrine precontraction. Repeated measures of one-way analysis of variance (ANOVA) were performed to analyze the repetitious concentration response curves, and when significancy was determined, post hoc analysis was carried out using Bonferroni test. Nonrepetitious data (E_max_, EC_50_, Hyc, TC, TG, Blood glucose) were evaluated by using nonparametric Kruskall-Wallis test. For all results a *P* value of <.05 was accepted to be significant.

## 3. Results

### 3.1. Metabolic Parameters

Body weight, blood glucose concentrations, serum total cholesterol, and triglyceride levels were presented in Table 1. Induction of diabetes with STZ resulted in a significant decrease in body weight and a significant increase in blood glucose levels compared to control rats. FF treatment affected neither high glucose levels nor weight loss in diabetic animals. Although diabetes was associated with significant increases in total cholesterol and triglyceride levels, FF treatment did not alter serum lipid levels significantly.

### 3.2. Vascular Functional Studies

#### 3.2.1. Contractile Responses

**Figure 1:**
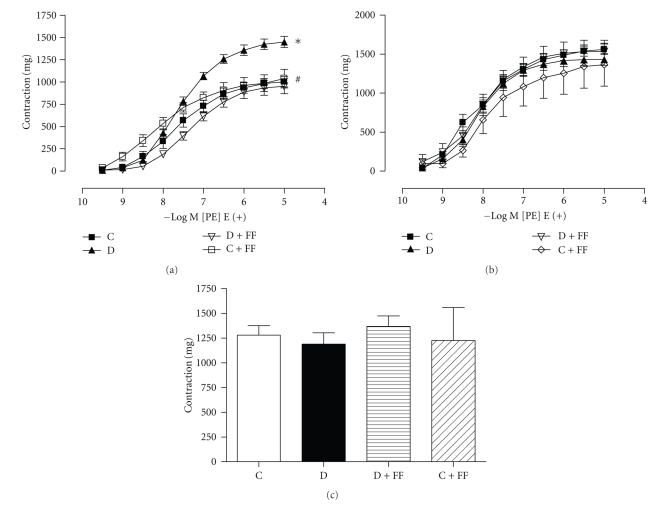
Concentration-dependent contraction to phenylephrine (PE) in endothelium-intact [E(+)] (a) and endothelium-denuded [E(−)] (b) aortic rings and contractions induced by a single concentration of KCl (120 mM) (c) in endothelium-denuded aortic rings. C: control; D: diabetes mellitus; FF: fenofibrate. *P < .05 versus C; ^#^ *P* < .05 versus D.

PE (1 nM–10 *μ*M) induced concentration-dependent contractile responses in the endothelium-intact aortic rings of all rats (Figure 1(a)). Contractility of PE was significantly increased in diabetic animals when compared to control group. However, FF provided a significant decrease in PE contractility in the diabetic group (Figure 1(a)). In the endothelium-denuded aortic rings, no significant changes in the PE contractility were observed between the experimental groups (Figure 1(b)). In addition, contractile responses to KCl were similar in all experimental groups (Figure 1(c)).

#### 3.2.2. Vasorelaxant Responses

**Figure 2:**
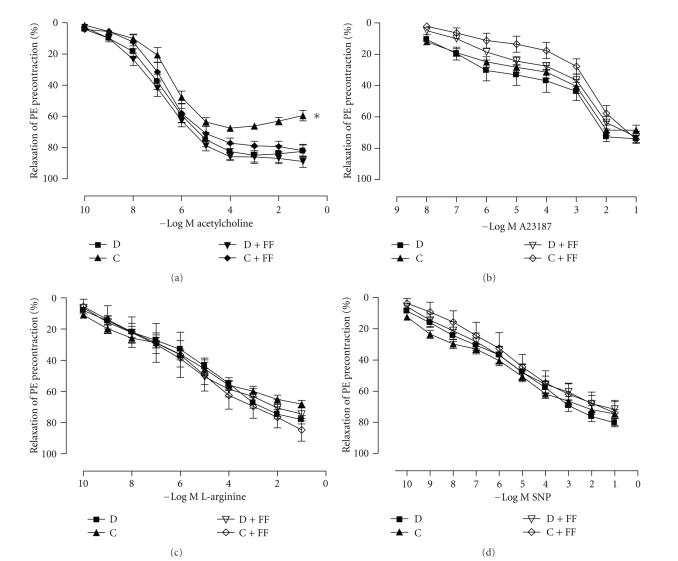
Relaxant responses to acetylcholine (ACh) (a), calcium ionophore A23187 (b), L-arginine (c) and sodium nitroprusside (SNP) (d) in the endothelium-intact aortic rings of control (C), and diabetic (D) rats treated with/without fenofibrate (FF). Values are mean ± SEM of data obtained from 10 rats in each group. *P < .05 versus C.

Endothelium-dependent relaxant response to ACh was significantly impaired in nontreated diabetic aorta (Figure 2(a)) without a significant difference in the *p*D_2_ value (Table 2). FF treatment restored the impaired relaxations to ACh and increased E_max  _ in the diabetic rats (Table 2). Concentration-dependent relaxant responses to A23187 (Figure 2(b)), L-arginine (Figure 2(c)), and SNP (Figure 2(d)), as well as *p*D_2_ and E_max  _ values (Table 2), did not show any significant differences between groups.

#### 3.2.3. Effect of L-NAME on Aortic Rings

**Figure 3:**
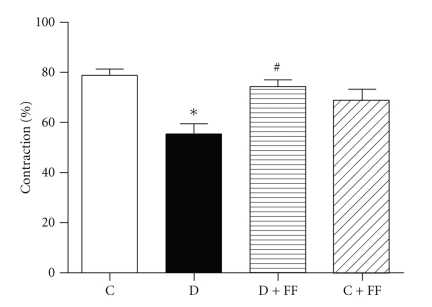
Contractile responses to NG-Nitro-L-arginine methyl ester (L-NAME) (100 *μ*M) in the endothelium-intact rings of control (C), and diabetic (D) rats treated with/without fenofibrate (FF). Responses were evaluated by calculating the ratio of additional contractions induced by L-NAME to the precontraction elicited by phenylephrine given at median effective concentration (EC_50_). The contractile responses obtained by L-NAME indicate the production of nitric oxide as a product of basal endothelial NO synthase activity. Values are mean ± SEM of data obtained from 10 rats in each group. *P < .05 versus C; ^#^ *P* < .05 versus D.

L-NAME was added to endothelium-intact aortic rings after precontracting the rings with the EC_50_ concentration of PE. L-NAME responses were significantly impaired in the nontreated diabetic group; however, FF brought in a significant improvement in these responses (Figure 3).

### 3.3. Antioxidant and Anti-Inflammatory Parameters

Erythrocyte-SOD and erythrocyte-catalase levels were markedly lower in the diabetic group than in the control group, and FF treatment caused a significant increase in these parameters (Table 3). Although liver-SOD levels were decreased, liver-catalase levels remained unchanged in the diabetic group. FF treatment significantly increased liver-SOD levels (Table 3).

In contrast, TBARS values in both erythrocytes and livers of the diabetic group were significantly higher than in the control group (Table 1). Although FF significantly lowered the erythrocyte-TBARS levels, it had no effect on the liver-TBARS levels. Aorta TBARS level was significantly increased in the diabetic group, and FF treatment markedly prevented the increased aortic TBARS level in diabetic group (Table 3).

Serum TNF-*α* and aortic MPO levels were significantly higher in the diabetic group than in the control group (Table 3). FF treatment decreased the high MPO levels in the diabetic rats without a significant effect on the TNF-*α* values.

### 3.4. Homocysteine Levels

Serum total Hcy levels were markedly decreased in diabetic rats when compared to control group (Table 3). FF treatment did not affect Hcy levels in diabetic rats; however, chronic administration of FF significantly increased Hcy levels in control rats.

## 4. Discussion

Data obtained in the present study reveal that in STZ-induced diabetic rats, chronic administration of FF improves endothelium-dependent relaxation and increases basal NO production in the aorta, enhances the levels of antioxidant enzymes such as SOD and catalase, ameliorates the abnormal TBARS levels, and finally, prevents the increased tissue MPO levels without a significant change in serum levels of total cholesterol and triglyceride. FF is one of the major drugs used in the treatment of dyslipidemia, and it has recently been reported that FF decreases serum levels of cholesterol and triglyceride in STZ-induced diabetic rats [22] and it produces a considerable decrease in serum triglyceride levels, a moderate reduction in LDL cholesterol levels, and a significant enhancement in HDL cholesterol concentrations in a model of diabetic dyslipidemia [23]. All these effects of FF have been attributed to the activation of PPAR-*α* by FF. Contrarily, we failed to show any beneficial effect of FF on the increased total cholesterol and triglyceride levels in diabetic rats; however FF prevented the diabetes-induced impairment in the endothelium-dependent relaxation. Although FF treatment is expected to reduce lipid levels, controversial studies also exist presenting that FF treatment does not affect plasma triglyceride and total cholesterol concentrations at doses up to 300 mg kg^−1^ day^−1^ in normoglycemic rats [24]. Moreover, another fibric acid derivative, bezafibrate, has been reported to affect none of the increased lipid levels (namely, total cholesterol, LDL, and triglyceride) in hyperglycemic rats at 30 mg kg^−1^ day^−1^ dose [25]. Our findings indicate that mechanisms other than the inhibition of high circulating lipids such as triglycerides and total cholesterol and the correction of dyslipidemia may involve in FF-induced restoration of endothelial dysfunction. 

It has been reported in various studies that increased oxidative stress, production of proinflammatory cytokines such as TNF-*α* and IL-6, leukocyte-mediated vascular injury with MPO, and hyperhomocysteinemia can be accepted as the crucial mechanisms responsible for the pathogenesis and progression of diabetic tissue damage [26–28]. Reduction in the acetylcholine-induced endothelium-dependent relaxation of diabetic rats in the present study is compatible with the results of several other studies [29–31]. In our study, induction of diabetes resulted in a marked decrease in acetylcholine response without a significant change in the *p*D_2_ value, indicating that the sensitivity of the diabetic aortic tissue to acetylcholine was preserved. Furthermore, endothelium-dependent relaxant responses to neither calcium ionophore A23187 nor L-arginine were found to be impaired. Even sodium-nitroprusside-induced endothelium-independent relaxation was preserved in diabetic aorta. These findings suggest that the vascular damage in our animal model is limited to the endothelium *per se*; however, the stimulated activation of eNOS seems not to be impaired nor does the utilization of the precursor L-arginine. In this case, it seems likely that endothelial dysfunction may be limited to the impairment in the homeostatic balance maintained by basal NO release. Indeed, PE contractility in endothelium-intact aorta was enhanced in diabetic vessels, and rings from diabetic animals responded to L-NAME with significantly greater tension, indicating a significant decrease of basal NO release in these vessels.

Enhanced contractility and decreased ACh responses may be associated with the deficient basal endothelial activity, besides, increased oxidative stress due to excessive production of oxygen-free radicals and decreased antioxidant defense systems may also involve in the process [32]. Oxidative degradation of lipids is a well-defined mechanism of cellular damage caused by excessive production of ROS, and TBARS is the most widely employed assay used to determine lipid peroxidation. Today, it is well known that TBARS level is increased in both plasma and aortic tissue of experimental diabetic models [33, 34]. In the present study, we have demonstrated that enhanced aortic, erythrocyte, and liver levels of TBARS in diabetic rats accompany the defective vascular endothelial function. In addition, SOD and catalase are known to be the most important enzymes in the antioxidant defence system of the body. The major function of SOD is to catalyze the conversion of superoxide anion radicals to hydrogen peroxide in order to reduce their toxic effects [34]. On the other hand catalase is responsible for the removal of intracellular hydrogen peroxide produced by SOD. Some *in vitro* studies have shown that ertyhrocyte antioxidant defences protect endothelial cells against oxidant-induced damage [35, 36]. In our study, we have observed that diabetic rats have decreased SOD levels both in the liver tissue and in the erythrocytes. Similarly, erythrocyte catalase levels were diminished without a significant change in the liver catalase levels. 

The mechanisms of the beneficial effects of FF on vascular function have not been fully understood yet. However, direct activation of PPAR-*α* in the arterial wall, correction of lipid abnormalities, and increment in the formation, availability, and action of NO have all been postulated. There is only one study published so far claiming that FF increases acetylcholine response in STZ-induced diabetic animals and reduces oxidative stress [22]. Data obtained in our study also confirm these vascular effects of FF.

We have also questioned the potential inflammatory mechanisms of diabetic vasculopathy and among them are the aortic MPO and TNF-*α* levels. Myeloperoxidase is a leukocyte-derived heme protein. Recent studies have shown that MPO plays an important role in endothelial dysfunction [27]. Plasma MPO levels are shown to be increased both in type 1 and type 2 diabetic patients [37]. An important consequence of MPO activity is the consumption of NO and thereby induction of endothelial dysfunction. The enzyme MPO can convert NO into nitrating oxidants, which are potential inflammatory mediators in cardiovascular diseases [37]. Compatible with up-to-date data, we have showed that aortic MPO levels are significantly increased in diabetic rats when compared to the control group. Evidence from recent studies suggest that TNF-*α* impairs endothelium-dependent and NO-mediated vasodilatation in various vascular beds such as rat thoracic aorta, coronary arteries, and carotid arteries [38]. The increased TNF-*α* expression induces the production of ROS, leading to endothelial dysfunction in diabetes. Besides TNF-*α* appears to decrease the bioavailability of NO by diminishing its production and enhancing its removal. Indeed, serum TNF-*α* levels in diabetic rats have been noted to increase in our study. FF treatment successfully prevented the diabetes-induced increase in aortic MPO levels; however it failed to affect the increased serum TNF-*α* levels. Controversial reports exist regarding the effect of FF on TNF-*α* levels. For example, Tian-Lun Yang has reported that FF reduces serum TNF-*α* levels of rats with LDL-induced endothelial dysfunction [39]. On the contrary, Choj has demonstrated that FF does not affect serum TNF-*α* levels in OLETF rats [40]. So far to our knowledge, no scientific reports exist using STZ-induced diabetic rat model to investigate the potential effects of FF on TNF-*α* levels, and we claim that FF has no effect on serum TNF-*α* levels. 

Despite its favourable effects on diabetic vascular dysfunction, FF treatment may result in elevation of serum total Hcy, which is known to induce oxidative stress and endothelial dysfunction [12]. Folate status has been associated with endothelial dysfunction in adolescents with type 1 diabetes, and elevated total Hcy is a risk factor for vascular disease in the nondiabetic population [41]. It is well known that FF increases total Hcy levels by PPAR-*α* mediated mechanism although it dose not affect unbound Hyc levels in rat serum [13]. In comparison to human beings, metabolism of Hcy is completely different in rats, and there is an extensive intrarenal Hcy metabolism in rats [42]. We have found decreased total Hcy levels in the serum of diabetic rats. It is probable that excessive protein loss due to diabetic nephropathy may cause a decrease in the protein-bound fraction of Hcy and the free form undergoes excessive metabolism resulting in a decrease in the total Hcy levels in diabetic rats. Indeed, our finding confirms to a previous report suggesting that serum total Hcy levels are found to be decreased in STZ-induced diabetic rat model [42]. In our study, FF did not affect serum total Hcy levels in the diabetic group, whereas it increased total Hcy levels in the control group probably through PPAR-*α* mediated mechanism; however the increase in total Hcy levels in FF-treated control rats did not impair the endothelial function. Therefore, we assume that fenofibrate-induced hyperhomocysteinemia does not involve in the pathogenesis of diabetes-induced endothelial dysfunction.

FF dose in the current study was selected from previous studies on rats [43]. It should be noted that this dose was much higher than those clinically used in the treatment of dyslipidemia (100–250 mg day^−1^). Additionally, it should be reminded that this is an *ex vivo*, but not an *in vivo*, study which is performed on the large, but not small, arteries of STZ-diabetic rats. Therefore, any potential antioxidant activity of FF requires to be investigated in well-conducted clinical trials performed on humans. Indeed, in humans, fibrate therapy has been reported to be associated with decreased levels of biomarkers of endothelial dysfunction in a few, but not all, studies presenting some clue on the preventory effect of fenofibrate in the development of diabetic microangiopathy [44–48]. Moreover, supplementation of FF treatment with antioxidants like coenzyme Q10 has been reported to have favourable vascular effects in the forearm microcirculation of dyslipidaemic type 2 diabetic patients, due to increase in the bioactivity of and/or responses to endothelium-derived relaxing factors, including NO, and this may entail synergistic stimulation of peroxisome proliferator-activated receptor [49].

In conclusion, treatment with FF produces a significant improvement in the endothelial dysfunction in STZ-induced diabetic rats without a significant effect on the serum total cholesterol and triglyceride levels. This effect of FF seems to be related to its potential antioxidant activity.

##  Conflict of Interests

The authors declare that there is no conflict of interests associated with this paper.

## Figures and Tables

**Table 1:** Metabolic parameters for control (C) and diabetic (D) rats treated with/without fenofibrate (FF).

	Body Weight (g)	Blood glucose (mg/dl)	Total cholesterol (mg/dl)	Triglyceride (mg/dl)
C	243.5 ± 7.0	122.7 ± 6.7	50.5 ± 2.2	65.9 ± 4.3
D	183.8 ± 5.9*	359.2 ± 24.9*	64.4 ± 4.7*	119.8 ± 9.9*
C + FF	250.5 ± 5.2	117.7 ± 5.4	63.7 ± 4.0	90.0 ± 9.2
D + FF	182.6 ± 9.6*	378.5 ± 42.6*	68.2 ± 4.3*	81.7 ± 11.5

Data are expressed as mean ± SEM, *n* = 10, *P < .05 versus C.

**Table 2:** E_max_ and *p*D_2_ values for control and diabetic rats.

	E_max _				*p*D_2_			
	C	D	D + FF	C + FF	C	D	D + FF	C + FF
Phenylephrine E(+)	1002 ± 57	1449 ± 61*	952 ± 83^#^	1038 ± 103	7.09 ± 0,13	7.10 ± 0,61	6.83 ± 0,81	7.71 ± 0.17
Phenylephrine E(−)	1562 ± 244	1432 ± 237	1534 ± 437	1390 ± 711	7.71 ± 0.51	7.66 ± 0.33	7.59 ± 0.36	7.85 ± 0.43
Acetylcholine	85.00 ± 0.30	67.63 ± 0.23*	89.08 ± 0.35^#^	82.00 ± 0.35	7.34 ± 0.03	7.26 ± 0.05	7.44 ± 0.04	7.33 ± 0.06
A23187	74.08 ± 0.29	68.5 ± 0.20	72.88 ± 0.33	74.37 ± 0.31	6.73 ± 0.09	6.72 ± 0.04	6.37 ± 0.06	6.14 ± 0.06
L-Arginine	78.21 ± 0.20	68.2 ± 0.20	74.70 ± 0.36	84.75 ± 0.95	5.66 ± 0.05	5.73 ± 0.04	5.98 ± 0.07	5.79 ± 0.19
Sodium nitroprusside	80.34 ± 0.22	74.69 ± 0.16	71.73 ± 0.36	74.06 ± 0.74	6.75 ± 0.06	6.80 ± 0.03	6.94 ± 0.06	6.79 ± 0.12

Data are expressed as mean ± SEM, *n* = 10.

E_max  _ is defined as the ratio of phenylephrine precontraction to maximum relaxation of acetylcholine, L-arginine, sodium nitroprusside, and calcium inonophore A23187.

*p*D_2_ is defined as −log EC_50_ concentration of drugs.

C: control; DM: diabetes mellitus; FF: fenofibrate; E(+): endothelium-intact aorta; E(−): endothelium-denuded aorta.

*P < .05 versus C, ^#^ *P* < .05 versus DM.

**Table 3:** Metabolic data for control and diabetic groups. Data are expressed as mean ± SEM of data obtained from 10 rats in each group. C: control; D: diabetes mellitus; FF: fenofibrate; TBARS: thiobarbituric acid reactive substances; MPO: myeloperoxidase; SOD: superoxide dismutase; TNF-*α*: tumor necrosis factor-alpha. *P < .05 versus C; ^#^ *P* < .05 versus DM.

	C	D	D + FF	C + FF
Erythrocyte SOD (U/g hemoglobin)	2433 ± 152	1592 ± 166*	2583 ± 316^#^	2514 ± 430
Erythrocyte Catalase (U/g hemoglobin)	5767 ± 418	4233 ± 196*	5552 ± 530^#^	5045 ± 718
Liver SOD (U/mg protein)	10.86 ± 1.21	5.34 ± 1.17*	9.78 ± 0.42^#^	9.67 ± 0.89
Liver Catalase (U/mg protein)	11.95 ± 1.09	9.32 ± 0.42	10.69 ± 0.89	11.32 ± 1.24
Erythrocyte TBARS (nmol/g hemoglobin)	536.84 ± 14.60	676.09 ± 14.67*	548.21 ± 46.33^#^	498.57 ± 67.82
Liver TBARS (nmol/mg protein)	16.37 ± 0.50	19.33 ± 0.62*	17.97 ± 0.88	16.92 ± 0.56
Aortic TBARS (nmol/mg protein)	1.029 ± 0.16	1.65 ± 0.25*	0.83 ± 0.19^#^	1.01 ± 0.11
Aortic MPO (U/g protein)	49.22 ± 2.80	87.36 ± 11.81*	13.29 ± 1.75*^,#^	15.43 ± 3.32*^,#^
Serum TNF-*α* (pmol/L)	3.44 ± 0.58	14.66 ± 5.43*	16.79 ± 4.84*	3.82 ± 0.97
Serum Homocysteine (*μ*mol/L)	9.07 ± 0.36	5.48 ± 0.34*	4.98 ± 0.39*	11.02 ± 0.61*
